# Exploring sensory phenotypes in autism spectrum disorder

**DOI:** 10.1186/s13229-021-00471-5

**Published:** 2021-10-12

**Authors:** Nichole E. Scheerer, Kristina Curcin, Bobby Stojanoski, Evdokia Anagnostou, Rob Nicolson, Elizabeth Kelley, Stelios Georgiades, Xudong Liu, Ryan A. Stevenson

**Affiliations:** 1 Brain and Mind Institute, Western University, 1151 Richmond St, London, ON N6A 3K7 Canada; 2 Department of Psychology, Western University, 1151 Richmond St, London, ON N6A 3K7 Canada; 3 Faculty of Social Science and Humanities, University of Ontario Institute of Technology, 2000 Simcoe Street North, Oshawa, ON L1H 7K4 Canada; 4 Holland Bloorview Kids Rehabilitation Hospital, 150 Kilgour Rd, East York, ON M4G 1R8 Canada; 5 Department of Psychiatry, Western University, 800 Commissioners Road East, B8-026, London, ON N6A 5W9 Canada; 6 Department of Psychology, Queens University, 62 Arch St., Kingston, ON K7L 3N6 Canada; 7 Department of Psychiatry, Queens University, 62 Arch St., Kingston, ON K7L 3N6 Canada; 8 McMaster University, 1280 Main St W, Hamilton, ON L8S 4L8 Canada; 9 Queens University, 99 University Ave, Kingston, ON K7L 3N6 Canada; 10 Department of Psychiatry, Western University, 1151 Richmond St, London, ON N6A 3K7 Canada

**Keywords:** Sensory processing, Sensory phenotypes, Cluster analysis, Autism spectrum disorder, Adaptive behaviour, Social communication, Restrictive and repetitive behaviours, ADHD traits, OCD traits

## Abstract

**Background:**

Atypical reactions to the sensory environment are often reported in autistic individuals, with a high degree of variability across the sensory modalities. These sensory differences have been shown to promote challenging behaviours and distress in autistic individuals and are predictive of other functions including motor, social, and cognitive abilities. Preliminary research suggests that specific sensory differences may cluster together within individuals creating discrete sensory phenotypes. However, the manner in which these sensory differences cluster, and whether the resulting phenotypes are associated with specific cognitive and social challenges is unclear.

**Methods:**

Short sensory profile data from 599 autistic children and adults between the ages of 1 and 21 years were subjected to a K-means cluster analysis. Analysis of variances compared age, adaptive behaviour, and traits associated with autism, attention-deficit and hyperactivity disorder, and obsessive and compulsive disorder across the resultant clusters.

**Results:**

A five-cluster model was found to minimize error variance and produce five sensory phenotypes: (1) sensory adaptive, (2) generalized sensory differences, (3) taste and smell sensitivity, (4) under-responsive and sensation seeking, and (5) movement difficulties with low energy. Age, adaptive behaviour, and traits associated with autism, attention-deficit and hyperactivity disorder, and obsessive and compulsive disorder were found to differ significantly across the five phenotypes.

**Limitations:**

The results were based on parent-report measures of sensory processing, adaptive behaviour, traits associated with autism, attention-deficit and hyperactivity disorder, and obsessive and compulsive disorder, which may limit the generalizability of the findings. Further, not all measures are standardized, or psychometrically validated with an autism population. Autistic individuals with an intellectual disability were underrepresented in this sample. Further, as these data were obtained from established records from a large provincial database, not all measures were completed for all individuals.

**Conclusions:**

These findings suggest that sensory difficulties in autistic individuals can be clustered into sensory phenotypes, and that these phenotypes are associated with behavioural differences. Given the large degree of heterogeneity in sensory difficulties seen in the autistic population, these sensory phenotypes represent an effective way to parse that heterogeneity and create phenotypes that may aid in the development of effective treatments and interventions for sensory difficulties.

**Supplementary Information:**

The online version contains supplementary material available at 10.1186/s13229-021-00471-5.

## Introduction

Autism spectrum disorder (ASD) is a neurodevelopmental condition characterized by persistent deficits in social communication and interaction and restricted, repetitive patterns of behaviour, interests, or activities [1]. Atypical reactions to the sensory environment are frequently reported in autistic individuals, with a high degree of variability across individuals and sensory modalities [2–6]. As our everyday lives are spent functioning in complex sensory environments, these sensory processing differences have been shown to promote challenging behaviours and distress in autistic individuals. In addition, these sensory differences have been shown to be predictive of other functions including cognitive, social, and motor abilities [7–9]. Given the relevance of sensory processing abilities to the diagnosis and support of autistic individuals, hyper- or hypo-reactivity to sensory input or an unusual interest in sensory aspects of the environment was added as core diagnostic features of ASD (DSM-5; [1]).

With this addition to the DSM-5, there has been considerable research into sensory processing differences. As a whole, the autistic population shows sensory processing differences across all sensory domains, however, these differences are idiosyncratic when considering the different sensory modalities within an individual [2–6]. Many of these recent studies have used the short sensory profile (SSP; [10]), which assesses sensory processing abilities across seven subscales including tactile sensitivity, taste and smell sensitivity, movement sensitivity, under responsivity and sensation seeking, auditory filtering, low energy and weakness, as well as visual and auditory sensitivity. Clustering techniques have been adopted in an attempt to parse heterogeneity across these domains and describe sensory phenotypes. Clustering involves grouping individuals with similar sensory processing abilities together in such a way that individuals in the same cluster have more similar sensory processing abilities to each other than individuals in other clusters. The resultant clusters can be thought of as sensory phenotypes, or distinct patterns of sensory processing abilities that commonly co-occur together.

A systematic review on this topic indicates that these studies have yielded between three- and five-cluster solutions [11]. Applying cluster analyses to SSP data typically yields a *sensory adaptive* phenotype that describes autistic individuals with mostly typical sensory processing, and a *generalized sensory differences* phenotype that describes autistic individuals who have difficulties across all of the sensory domains [4–6, 12, 13]. In addition to these phenotypes, there have been varied descriptions of phenotypes that exhibit other distinct patterns of sensory difficulties, including a *sensory moderate* phenotype [12], a *taste and smell sensitivity* phenotype [4–6], an *under-responsive and sensory seeking* phenotype [5], a *tactile and movement difficulties* phenotype [5], and a *movement difficulties with low energy* phenotype [6]. Although the previously identified phenotypes offer insight into sensory processing in autism, replication of these findings using a much larger sample size is required to determine their reliability. Thus, the first aim of the current study is to investigate patterns in sensory processing abilities in a large sample of autistic children and adults in order to determine the best number of sensory phenotypes to describe their sensory processing abilities.

The identification of sensory phenotypes has practical and clinical applications, as sensory issues have been shown to be predictive of the cognitive and social development of autistic individuals. Sensory processing abilities have been related to adaptive functioning [4, 14], autism traits [4, 14], and traits associated with co-occurring conditions such as attention-deficit/hyperactivity disorder (ADHD) and obsessive compulsive disorder (OCD; [14, 15]). Greater specificity in terms of how sensory processing across different sensory domains relates to differences in functional behaviours is required before informing interventions and support strategies. Given this, the second aim of the current study is to identify relations between sensory processing differences and adaptive functioning, autism traits, and traits related to commonly co-occurring neurodevelopmental disorders including ADHD and OCD.

Sensory processing differences have also been linked to demographic factors such as age, IQ, and sex assigned at birth. The relationship between sensory processing and age is currently unclear, with reports that sensory hypersensitivity decreases with age [16, 17], that sensory seeking and reactivity increase with age [2, 16], or that there is no relationship between age and sensory processing [5, 12, 14, 18, 19]. Similar to age, some researchers report that IQ differs as a function of sensory processing abilities [6], while others report no relationship [7, 14, 20]. While sensory processing abilities have been found to differ across autistic children [21, 22] and adults’ [23, 24] sex assigned at birth, these differences have not been found across different sensory phenotypes [14]. However, given the uneven sex ratios in autism coupled with small samples, appropriately powered investigations are sorely lacking. Given these findings, it may also be important to consider age, IQ, and sex assigned at birth when examining sensory processing differences in autistic individuals.

The current study used cluster-based analyses on one of the largest samples of autistic children and young adults to date to explore sensory-based phenotypes of practical significance. We aim to not only resolve the ambiguity as to the best number of sensory phenotypes to describe sensory processing in autistic children and young adults, but also to extend these findings by exploring how these sensory phenotypes are related to adaptive functioning, and autism, ADHD and OCD traits, and demographic factors including age, IQ, and sex.

## Methods

### Participants

Data from 599 participants (Age_(M,SD)_ = 10.00, 4.44; 472 male, 127 female) were extracted from the Province of Ontario Neurodevelopmental Disorder (POND) Network’s database (https://pond-network.ca). Participants were included if they had a diagnosis of ASD, Autism, Asperger’s, or Pervasive Developmental Disorder Not Otherwise Specified and a completed short sensory profile (SSP; [10]). These diagnoses were made by general and paediatric physicians, psychiatrists, developmental behavioural paediatricians, and psychologists. Diagnoses were confirmed using the autism diagnostic observation schedule (ADOS; [25]) and autism diagnostic interview (ADI-R; [26]) administered by reliable examiners. Individuals with comorbid diagnoses were not excluded since there is evidence that autism has significant diagnostic overlap with other diagnoses [27]. Common comorbidities included ADHD (19.03%), anxiety disorders (14.19%), intellectual disabilities (8.51%), and learning disorders (11.02%). Participants and their parents or caregivers also completed a range of measures to assess the participant’s IQ, sensory processing abilities, adaptive behaviours, autistic traits, ADHD traits, and OCD traits. Study procedures were approved by the Research Ethics Board at Western University, and ethical approval was also obtained at each data collection site, in accordance with the World Medical Association’s 2013 Declaration of Helsinki.

### Materials

Cognitive abilities were tested using standardized measures of intelligence that were appropriate for the participant’s age and developmental level. Wechsler tests, the Wechsler Abbreviated Scales of Intelligence—Second Edition ([28]; *n* = 319), the Wechsler Intelligence Scale for Children Version 4 ([29]; *n* = 21), and the Wechsler Preschool and Primary Scale of Intelligence Version 4 ([30]; *n* = 14), were prioritized when individuals were of the appropriate age, were verbally fluent, and there was sufficient time. The Stanford–Binet Intelligence Scale ([31]; *n* = 116), the Mullen Scales of Early Learning ([32]; *n* = 45), and the Leiter International Performance Scale Version 3 ([33]; *n* = 6) were used for those who were too young or unable to complete the Wechsler tests. IQ data for 78 participants were not available.

Short sensory profile. The short sensory profile is a well-validated, 38-item parent report questionnaire designed to measure behaviours associated with abnormal responses to sensory information in children between the ages of 3 and 10 years [34–36]. The questionnaire consists of 7 subscales including tactile sensitivity (7 items), taste/smell sensitivity (4 items), movement sensitivity (3 items), under-responsive/seeks sensation (7 items), auditory filtering (6 items), low energy/weak (6 items) and visual/auditory sensitivity (5 items). Parents respond to each question on a five-point Likert scale (always (100% of the time) = 1, frequently (75% of the time) = 2, occasionally (50% of the time) = 3, seldom (25% of the time) = 4, or never (0% of the time) = 5) indicating the frequency with which their child displays the sensory behaviour. The SSP produces an unstandardized score with lower scores indicating greater sensory processing abnormalities. The SSP has been shown to have strong internal consistency in individuals with ASD (0.89; [36]) and is widely used in studies of sensory perception as it covers a wide range of sensory processing domains. While the SSP was initially developed on typically developing children, a confirmatory factory analysis has indicated that the seven-subscale structure is also appropriate for quantifying sensory processing in autistic children and young adults between the ages of 1 and 22 years [37].

Vineland Adaptive Behaviour Scales—Second Edition. The Vineland Adaptive Behaviour Scales—Second Edition (VABS-II; [38]) is a caregiver interview-based measure of a child’s personal and social skills and is often used as an adaptive behaviour measure in ASD. Each question is scored Usually = 2, Sometimes or Partially = 1, or Never = 0, with higher scores indicative of more adaptive behaviours. There are 4 domains and a total composite score that are all standardized based on a normative mean of 100 and a standard deviation of 15 for the given age. The behavioural domains include communication, daily living skills, socialization, and motor skills (for those 6 years of age and under). The internal consistency reliability of the domain and adaptive behaviour composite scores show Cronbach’s α’s ranging from 0.88 to 0.97 [38]. The daily living skills subscale has been shown to be the least confounded with other aspects of autism, such as cognitive ability [4]. Thus, the daily living skills subscale is considered the most suitable measure of adaptive functioning from the VABS-II for children with ASD.

Repetitive Behaviour Scale—Revised (RBS-R). The RBS-R [39], is a 43-item questionnaire administered to parents of children ages 6–17. The RBS-R aims to measure the breadth of repetitive behaviours in children and adolescents with ASD. The RBS-R consists of six subscales including: Stereotyped Behaviour, Self-injurious Behaviour, Compulsive Behaviour, Routine Behaviour, Sameness Behaviour, and Restricted Behaviour, that have no overlap of item content. Items are scored as behaviour does not occur = 0, behaviour occurs and is a mild problem = 1, behaviour occurs and is a moderate problem = 2, behaviour occurs and is a severe problem = 3. The RBS-R produces an unstandardized score, with total overall scores indicating the prevalence of more problematic behaviours. We also assessed repetitive behaviours using the four-factor structure consisting of Stereotypy, Self-Injury, Compulsions, and Ritualistic/Sameness subscales (see [40]). Cronbach’s alpha for these subscales indicates high internal consistency with alphas ranging from 0.8 to 0.92 [40].

Social Communication Questionnaire (SCQ)-Lifetime Form. The SCQ [41] is a 40-item parent questionnaire used to assess communication skills and social functioning in children who may have autism. The questionnaire considers lifetime characteristics across 3 domains of social relating, communication, and range of interests, which are assessed using yes/no responses. The SCQ produces an unstandardized score, with total scores above 15 suggesting that the individual is likely to be on the autism spectrum. The SCQ has high internal consistency (Cronbach’s alpha = 0.87; [41], and good discriminative validity when distinguishing between children with ASD and non-ASD diagnoses. The sensitivity of the SCQ is about 96%, while the specificity is about 80%, in samples of children without intellectual disability [41]. Note that while the SCQ contains questions pertaining to a child’s range of interests, given the questions are primarily social in nature, the SCQ was used as an index of autistic social behaviours.

Strengths and Weaknesses of Attention-Deficit/Hyperactivity Disorder Symptoms of Normal Behaviour Scale (SWAN; [42]). The SWAN is a 18-item caregiver questionnaire designed for children under the age of 18 years. The questionnaire includes scoring of both strengths and weaknesses associated with symptoms of ADHD. Each question is scored on a seven-point scale, with Far Below Average = 3, Below Average = 2, Somewhat Below Average = 1, Average = 0, Somewhat Above Average =  − 1, Above Average =  − 2, and Far Above Average =  − 3. The SWAN produces an unstandardized score, with higher scores indicate greater symptomatology. Two subscale scores can be produced, the inattention subscale, and the hyperactivity subscale. The SWAN has high internal consistency (Cronbach's *α* = 0.88), and reliability ranged from 0.72 to 0.90 (*M* = 0.82; [43]).

Toronto Obsessive–Compulsive Scale (TOCS). The TOCS [44] is a 21-item parent report questionnaire assessing obsessive–compulsive traits. Domains include cleaning/contamination, symmetry/ordering, counting/checking, rumination, superstition, and hoarding. The TOCS has excellent internal consistency (Cronbach's *α* = 0.94), with sensitivity and specificity analyses indicating that an unstandardized TOCS total score of greater than 0 successfully discriminates community-reported obsessive–compulsive disorder (OCD) cases from non-cases.

### Analysis

Statistical analyses were conducted using R (v. 4.0.2, Vienna, Austria) and the Statistical Package for the Social Sciences (SPSS v. 24, New York, New York, USA). Descriptive statistics were calculated for each of the measures. The SSP subscale scores were then converted to *z*-scores and submitted to *k*-means cluster analyses to determine patterns of sensory processing in this sample. A cluster analysis is an exploratory data analysis technique used to identify subgroups (or clusters) in a dataset that represent data points that are very similar to one another, yet distinct from data points in other clusters. The *k*-means algorithm clusters the data into a number, *k*, of predefined, distinct, and non-overlapping groups where each data point only belongs to one group. Data points are assigned to a particular cluster in such a way that the sum of the squared distance between all of the data points, and the mean of all the data points that belong to that cluster, is minimized [45]. Applying the *k*-means approach to the subscales of the SSP allowed us to examine how sensory processing differences cluster together, with each of the resulting clusters representing a distinct sensory phenotype. Based on a systematic review indicating 3–5 sensory phenotypes in autism, we tested *k*’s of 2–6 [11]. To determine the best-fit model, we used Bayesian Information Criteria (BIC; [46]), previous literature [2, 4–6, 12, 19, 33, 47–50]), and comparisons with behavioural clinical measures to help quantify the practical, real-world significance of these sensory phenotypes. Welch’s one-way analysis of variances (ANOVAs) assuming unequal variances with follow-up Games–Howell post hoc comparisons were used to compare SSP subscale scores across the sensory clusters. Chi-square tests were used to compare sex at birth across the clusters in each model solution, while Welch’s one-way ANOVAs with Games–Howell post hoc comparisons were conducted to compare IQ, adaptive functioning, ASD traits, ADHD traits, and OCD traits, across the clusters in each model solution.

## Results

Table 1 reports the descriptive results for key demographic and experimental measures. Mean scores on the tactile, taste/smell, movement, and visual auditory sensitivity subscales fell into the probable difference, while scores on the under-responsive/seeks sensation, auditory filtering, and low energy subscales fell into the definite difference in sensory processing function range when comparing the mean scores to normative data based on the performance of children without disabilities (*n* = 1037; [10]).

**Table 1:** Participant characteristics

	*n*	Mean	SD	Range
Age	599	10.00	4.44	1–21
*Sex assigned at birth*				
Male	472			
Female	127			
IQ-full	521	86.23	25.60	40–142
IQ-verbal	472	88.38	24.31	43–160
IQ-performance	492	89.89	25.72	42–160
*Short sensory profile*	*599*			
Tactile		26.51	5.79	9–35
Taste Smell		12.90	5.50	3–20
Movement		12.31	3.02	1–15
Under-responsive/seeks sensation		21.56	6.79	7–35
Auditory filtering		16.85	4.99	6–30
Low energy weak		22.47	7.19	0–30
Visual auditory		17.03	5.13	5–25
Total		129.69	24.69	54–190
*VABS-II*				
Communication	435	73.38	16.53	26–136
Daily living skills	434	71.94	15.39	25–125
Socialization skills	434	70.34	14.82	32–118
Motor skills	119	81.17	14.17	51–114
Adaptive behaviour	428	70.37	14.05	23–123
*SWAN*	*463*			
Inattention subscale		4.67	3.04	0–9
Hyperactive subscale		3.94	3.11	0–9
*TOCS*	410	− 14.37	25.67	− 63–45
*RBS*	567	30.32	19.40	1–92
Self-injury		2.99	3.67	0–20
Stereotypy		6.50	4.64	0–22
Ritualistic/sameness		14.72	9.58	0–43
Compulsions		4.32	4.40	0–24
*SCQ*	534	19.81	7.15	2–37

Abbreviation: *SSP* short sensory profile, *IQ* intelligence quotient, *VABS-II* Vineland Adaptive Behavioural Scales, *RBS-R* Repetitive Behaviour Scale—Revised, *SCQ* Social Communication Questionnaire, *TOCS* Toronto Obsessive–Compulsive Scale, *SWAN* Strengths and Weaknesses of Attention-Deficit/Hyperactivity Disorder Symptoms of Normal Behaviour Scale

### Patterns of sensory behaviour

**Fig. 1:**
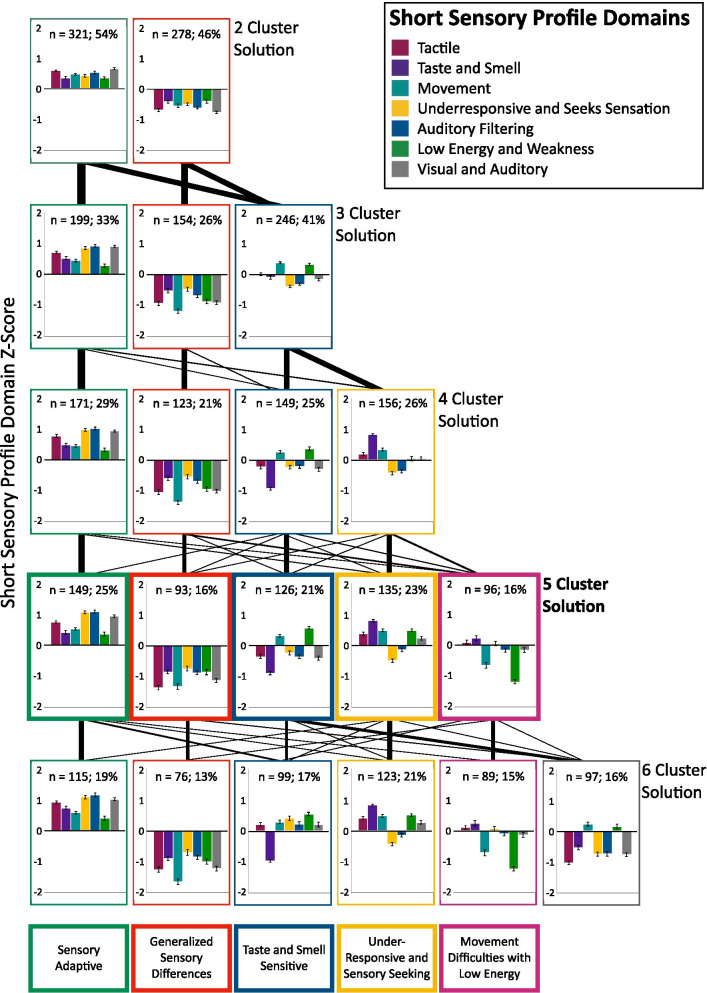
Short sensory profile domain Z-scores across the *k* 2–6 cluster solutions. Negative z-scores are indicative of increased sensory difficulties. Line weights between cluster solutions represent the number of participants remaining/changing clusters across solutions. Error bars indicate standard error of the mean

**Fig. 2:**
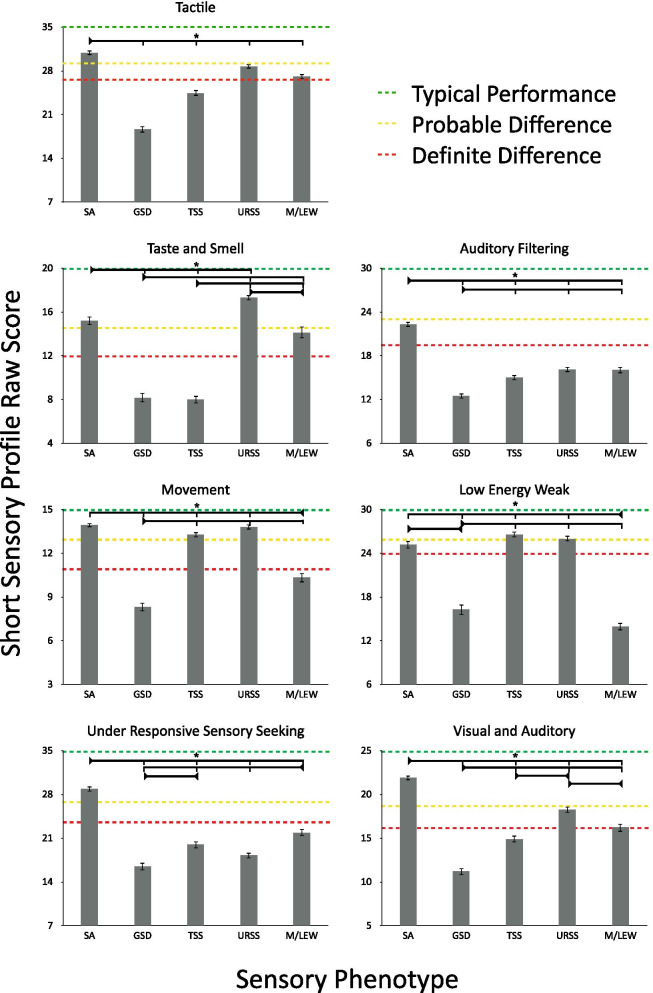
Short sensory profile domain raw scores across the five sensory phenotypes: sensory adaptive (SA), generalized sensory difference (GSD), taste and smell sensitive (TSS), under-responsive and sensory seeking (URSS), and movement difficulties with low energy. Error bars indicate standard error of the mean. Green (typical difference), yellow (probable difference), and red (definite difference) classification is based on a comparison with the performance of children without disabilities [10]

Results of the *k*-means cluster analyses conducted in R indicated that a five-cluster solution produced the best-fit model based on previous literature, BIC values, and consideration of the practical, real-world significance of the resultant sensory phenotypes. A bootstrapping technique was used to produce 100 iterations of the five-cluster solution to ensure the reliability of the selected model, and BIC values were examined (see Additional file 1). Starting with a *K* of 2, the *k*-means cluster analysis fit a model that clustered participants by high or low sensory processing abnormalities (see Fig. 1). With the addition of each successive cluster, the model produced a group of clusters that highlighted distinct patterns of sensory processing abnormalities. However, once the six-cluster model emerged, the new cluster failed to produce a highly differentiated pattern of sensory processing abnormalities. Given the pattern of the SSP subscale scores across the clusters in the five-cluster model, we classified cluster 1 as a sensory adaptive (SA) phenotype, cluster 2 as a generalized sensory differences (GSD) phenotype, cluster 3 as a taste and smell sensitivity (TSS) phenotype, cluster 4 as an under-responsive and sensory seeking (URSS) phenotype, and cluster 5 as a movement and low energy/weakness (M/LEW) phenotype (see Fig. 2).

One-way ANOVAs were conducted on the SSP subscale scores (*z*-scores: see Fig. 1, raw scores: see Fig. 2) using SPSS to determine whether the SSP subscales differed across the 5 phenotypes. All 7 subscales, tactile, taste/smell, movement, under-responsive/sensory seeking, auditory filtering, low energy/weak, and auditory filtering differed significantly across the 5 phenotypes (see Figs. 1, 2; Additional file 2 contains the full statistical analyses, while Additional file 3 contains correlations between all experimental variables). In addition, the internal consistency of the SSP was assessed, with Cronbach's *α* = 0.917 for the total score and Cronbach’s *α*’s ranging from 0.775 to 0.932 for the SSP subscales, indicating excellent internal consistency (see Additional file 2).

### Sensory phenotypes and demographic factors

#### Age

A one-way ANOVA indicated that age differed across the sensory phenotypes (F(4, 283.6) = 13.55, *p* < 0.001, est. *w*^2^ = 0.077; see Table 2). Post hoc comparisons indicate that participants with the TSS phenotype were significantly younger than participants with the SA (*t*(273(= − 5.26, *p* < 0.001, *d* = 0.63), GSD (*t*(199) =  − 4.85, *p* < 0.001, *d* = 0.66), and M/LEW (*t*(207) =  − 6.45, *p* < 0.001, *d* = 0.87) phenotypes (see Fig. 4). Participants with the URSS phenotype were also significantly younger than participants with the SA (*t*(280) =  − 2.79, *p* = 0.045, *d* = 0.33) and M/LEW (*t*(206) =  − 4.04, *p* < 0.001, *d* = 0.54) phenotypes.

**Table 2:** Descriptive and test statistics for measured variables

	SA M (SD)	GSD M (SD)	TSS M (SD)	URSS M (SD)	M/LEW M (SD)	Test statistic	Significant contrasts
Age	10.43 (4.86)	10.30 (4.09)	7.59 (4.11)	8.96 (4.05)	11.13 (4.00)	*F*(4, 283.6) = 13.55, *p* < 0.001, est. *w*^2^ = 0.077	TSS < SA, GSD, M/LEW; URSS < SA, M/LEW
IQ							
Full-scale	85.92 (25.57)	85.41 (19.97)	84.78 (27.61)	87.34 (28.84)	87.87 (23.45)	*F*(4, 245.5) = 0.25, *p* = 0.909, est. *w*^2^ = − 0.006	–
Verbal	87.49 (24.70)	87.62 (20.81)	88.61 (25.70)	88.20 (26.97)	90.51 (21.88)	*F*(4, 224.7) = 0.25, *p* = 0.909, est. *w*^2^ = − 0.006	–
Performance	89.24 (25.88)	85.98 (20.95)	91.91 (25.95)	93.03 (29.94)	88.33 (23.17)	*F*(4, 236.2) = 1.20, *p* = 0.313, est. *w*^2^ = 0.002	–
Sex	42F, 107 M	19F, 74 M	25F, 101 M	21F, 114 M	20F, 76 M	*x*^2^(4) = 7.109, *p* = 0.130, Cramer’s *V* = 0.055	–
VABS-II							
Adaptive behaviour composite	75.61 (13.89)	65.25 (11.91)	72.00 (11.78)	69.63 (15.29)	66.71 (14.43)	*F*(4, 203.5) = 8.62, *p* < 0.001, est. *w*^2^ = 0.007	SA > GSD, URSS, M/LEW; GSD < TSS
Communication skills	79.24 (16.37)	69.93 (13.96)	74.09 (15.46)	71.61 (18.20)	69.87 (16.15)	*F*(4, 209.0) = 5.58, *p* < 0.001, est. *w*^2^ = 0.040	SA > GSD, URSS, M/LEW
Daily living skills	77.32 (14.63)	65.83 (13.37)	74.12 (13.27)	72.16 (17.10)	67.43 (15.32)	*F*(4, 207.3) = 9.51, *p* < 0.001, est. *w*^2^ = 0.073	GSD < SA, TSS, URSS; M/LEW < SA, TSS
Socialization skills	75.70 (16.08)	64.59 (12.16)	71.19 (12.41)	69.71 (14.96)	68.23 (15.50)	*F*(4, 207.9) = 7.40, *p* < 0.001, est. *w*^2^ = 0.056	SA > GSD, URSS, M/LEW; TSS > GSD
Motor skills	87.63 (12.85)	76.92 (8.75)	81.23 (13.95)	81.55 (15.52)	66.00 (6.69)	*F*(4, 40.4) = 11.45, *p* = 0.001, est. *w*^2^ = 0.260	M/LEW < SA, TSS, URSS
RBS-R total score	16.88 (12.93)	48.55 (19.33)	37.25 (19.18)	26.02 (15.03)	30.81 (15.85)	*F*(4, 260.0) = 58.96, *p* < 0.001, est. *w*^2^ = 0.290	SA < GSD, TSS, URSS, M/LEW; GSD > TSS, URSS, M/LEW; TSS > URSS
Self-injury	1.47 (2.19)	5.30 (4.72)	3.31 (3.45)	2.86 (3.54)	2.93 (3.68)	*F*(4, 250.8) = 17.62, *p* < 0.001, est. *w*^2^ = 0.105	SA < GSD, TSS, URSS, M/LEW, GSD > TSS, URSS, M/LEW
Stereotypy	3.45 (3.08)	9.50 (4.59)	8.25 (4.70)	6.62 (4.24)	5.93 (4.25)	*F*(4, 258.5) = 43.76, *p* < 0.001, est. *w*^2^ = 0.232	SA < GSD, TSS, URSS, M/LEW, GSD, TSS > URSS, M/LEW
Compulsions	2.11 (2.70)	7.28 (5.52)	5.40 (4.57)	3.72 (3.43)	4.36 (4.31)	*F*(4, 252.3) = 25.79, *p* < 0.001, est. *w*^2^ = 0.149	SA < GSD, TSS, URSS, M/LEW, GSD > URSS, M/LEW, TSS > URSS
Ritualistic/sameness	8.88 (7.48)	23.31 (9.11)	18.12 (9.24)	11.58 (7.67)	15.41 (7.72)	*F*(4, 264.4) = 48.96, *p* < 0.001, est. *w*^2^ = 0.253	SA < GSD, TSS, URSS, M/LEW, GSD > TSS, URSS, M/LEW, URSS < TSS, M/LEW
SCQ	16.02 (6.41)	23.62 (6.97)	21.02 (5.99)	19.56 (7.42)	20.61 (6.78)	*F*(4, 253.2) = 18.90, *p* < 0.001, est. *w*^2^ = 0.118	SA < GSD, TSS, URSS, M/LEW; GSD > TSS, URSS, M/LEW
SWAN							
Inattention	3.05 (2.79)	5.69 (2.75)	4.38 (3.03)	5.40 (3.07)	5.24 (2.75)	*F*(4, 223.6) = 14.95, *p* < 0.001, est. *w*^2^ = 0.107	SA < GSD, TSS, URSS, M/LEW; TSS < GSD
Hyperactivity	2.27 (2.62)	4.99 (3.06)	4.30 (3.14)	4.66 (2.97)	3.98 (3.05)	*F*(4, 221.2) = 15.48, *p* < 0.001, est. *w*^2^ = 0.111	SA < GSD, TSS, URSS, M/LEW
TOCS	− 20.68 (26.25)	-6.14 (25.29)	− 11.55 (25.68)	− 19.72 (24.54)	− 10.23 (23.63)	*F*(4, 196.3) = 5.21, *p* < 0.001, est. *w*^2^ = 0.039	M/LEW > SA; GSD > SA, URSS

*SA* sensory adaptive, *GSD* generalized sensory differences, *TSS* taste and smell sensitivity, *URSS* under-responsive sensory seeking, *M/LEW* movement difficulties with low energy and weakness, *IQ* intelligence quotient, *VABS-II* Vineland Adaptive Behaviour Scales—Second Edition, *RBS-R* Repetitive Behaviours Scale—Revised, *SCQ* Social Communication Questionnaire, *SWAN* Strengths and Weakness of Attention-Deficit/Hyperactivity Disorder Symptoms of Normal Behaviour Scale, *TOCS* Toronto Obsessive Compulsive Scale. Contrasts are significant at *p* < 0.05

#### IQ

**Fig. 3:**
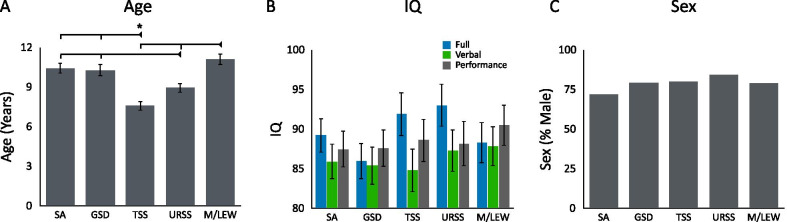
Age (**A**), IQ (**B**), and Sex (**C**) assigned at birth as a function of sensory phenotype (sensory phenotypes: sensory adaptive (SA), generalized sensory difference (GSD), taste and smell sensitive (TSS), under-responsive and sensory seeking (URSS), and movement difficulties with low energy (M/LEW). Error bars indicate standard error of the mean. *Note*: *indicates significance at *p* < 0.05

One-way ANOVAs indicated that there were no differences in IQ across sensory phenotypes for full-scale (*F*(4, 245.5) = 0.250, *p* = 0.909, est. *w*^2^ =  − 0.006), verbal (*F*(4, 224.7) = 0.251, *p* = 0.909, est. *w*^2^ =  − 0.006), and performance (*F*(4, 236.2) = 1.196, *p* = 0.313, est. *w*^2^ = 0.002) IQ (see Fig. 3).

#### Sex assigned at birth

A chi-squared analysis indicated that sex did not vary significantly across the phenotypes (*x*^2^(4) = 7.109, *p* = 0.130, Cramer’s *V* = 0.055; see Fig. 3).

### Sensory phenotypes and adaptive functioning

**Fig. 4:**
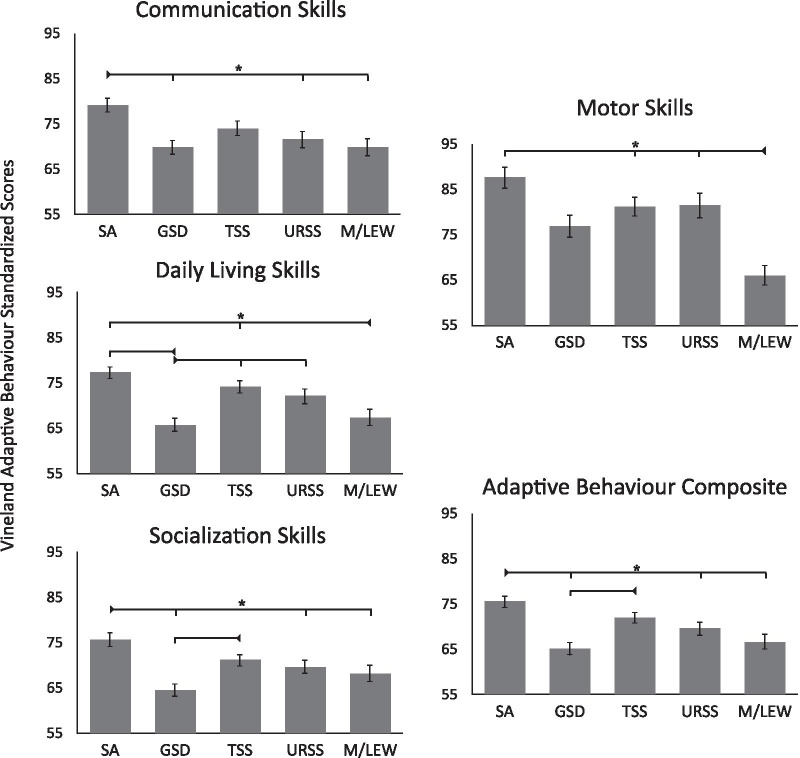
Adaptive behaviours as a function of sensory phenotype (sensory phenotypes: sensory adaptive (SA), generalized sensory difference (GSD), taste and smell sensitive (TSS), under-responsive and sensory seeking (URSS), and movement difficulties with low energy (M/LEW). Error bars indicate standard error of the mean. *Note*: *indicates significance at *p* < 0.05

One-way ANOVAs were conducted to determine whether the VABS-II adaptive behaviour scores differed across the sensory phenotypes (see Table 2 and Additional file 4 for full statistics). Overall, the Adaptive Behaviour Composite score, as well as the communication skills, daily living skills, socialization skills, and motor skills subscales all differed significantly across the sensory phenotypes (all ps < 0.001; see Fig. 4 and Additional file 4 for full statistics).

### Sensory phenotypes and ASD traits

**Fig. 5:**
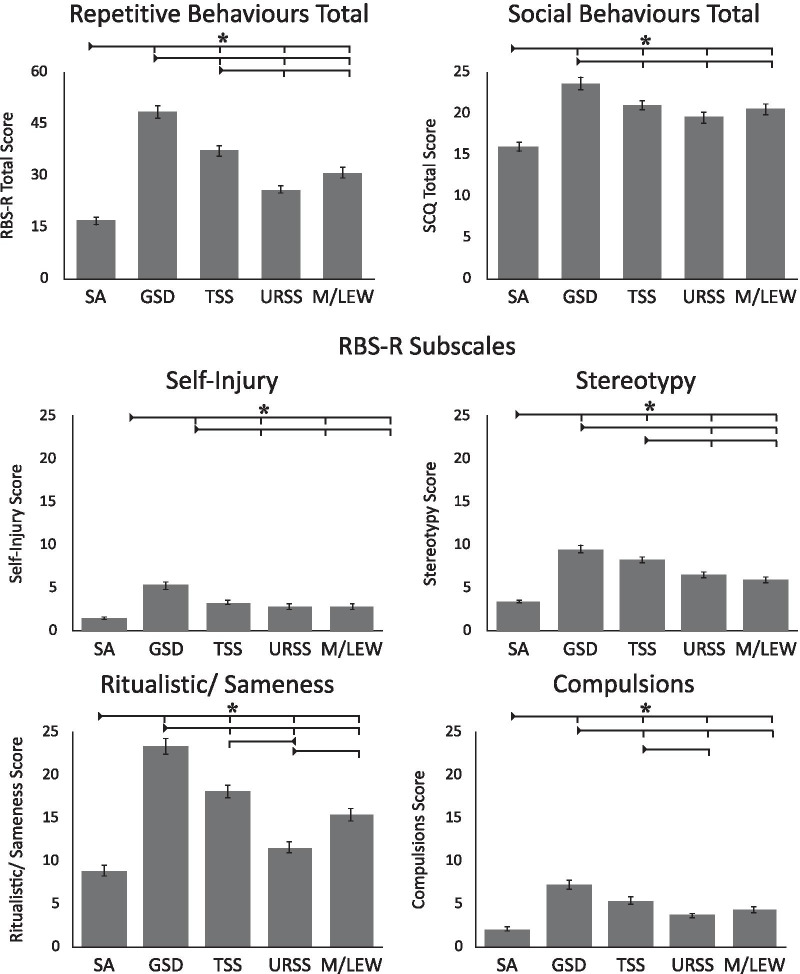
Repetitive behaviours, measured by the RBS, and social behaviours, measured by the SCQ, as a function of sensory phenotype. Error bars indicate standard error of the mean. Higher scores are indicative of more repetitive behaviours on the RBS, and more social difficulties on the SCQ. *Note*: *indicates significance at *p* < 0.05

A one-way ANOVA indicated that repetitive behaviours, measured by the RBS-R, were found to differ across sensory phenotypes (all ps < 0.001; see Table 2). The repetitive behaviours total score, as well as the self-injury, stereotypy, ritualistic/sameness, and compulsions subscales all differed across the sensory phenotypes (see Fig. 5; Additional file 5 for full statistics). In addition, the internal consistency of the RBS-R was assessed, with Cronbach's *α* = 0.934, indicating excellent internal consistency.

Autistic social behaviours, measured by the SCQ, were also found to differ across the sensory phenotypes (*F*(4, 253.2) = 18.90, *p* < 0.001, est. *w*^2^ = 0.118.; see Fig. 5 and Table 2). Participants with the SA phenotype had better social skills than participants with all other phenotypes (GSD: *t*(167) =  − 8.04, *p* < 0.001, *d* = 1.14; TSS: *t*(230) =  − 6.18, *p* < 0.001, *d* = 0.81; URSS: *t*(241) =  − 4.05, *p* < 0.001, *d* = 0.51; M/LEW: *t*(189) =  − 5.08, *p* < 0.001, *d* = 0.70). Participants with the GSD also had poorer social skills than participants with the TSS (*t*(164) = 2.72, *p* = 0.056, *d* = 0.40; marginal), URSS (*t*(186) = 4.01, *p* < 0.001, *d* = 0.56), and M/LEW (*t*(172) = 2.90, *p* = 0.034, *d* = 0.44) phenotypes. In addition, the internal consistency of the SCQ was assessed, with Cronbach's *α* = 0.852, indicating good internal consistency.

### Sensory phenotypes and ADHD traits and OCD traits

#### ADHD traits

**Fig. 6:**
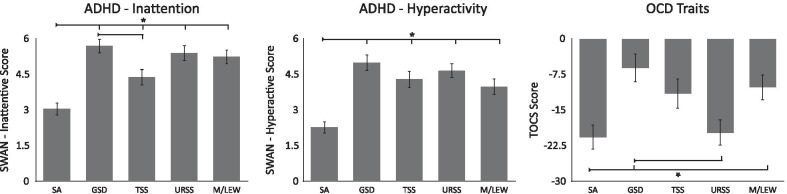
ADHD traits (inattention, hyperactivity) as measured by the SWAN, and OCD traits, as measured by the TOCS, as a function of sensory phenotype. Higher scores on the SWAN are indicative of more ADHD traits, while lower scores on the TOCS are indicative of more OCD traits. Error bars indicate standard error of the mean. *Note*: *indicates significance at *p* < 0.05

SWAN scores indicated that both *inattention* (*F*(4, 223.6) = 14.95, *p* < 0.001, est. *w*^2^ = 0.107) and *hyperactivity* (*F*(4, 221.2) = 15.48, *p* < 0.001, est. *w*^2^ = 0.111) differed across the sensory phenotypes (see Fig. 6 and Table 2). Participants with the SA phenotype had lower levels of inattention than those with the GSD (*t*(181) =  − 6.64, *p* < 0.001, *d* = 0.95), TSS (*t*(161) =  − 3.09, *p* = 0.020, *d* = 0.46), URSS (*t*(200) =  − 5.82, *p* < 0.001, *d* = 0.80), and M/LEW (*t*(187) =  − 5.57, *p* < 0.001, *d* = 0.79) phenotypes. Participants with the TSS phenotype also showed lower levels of inattention than those with the GSD (*t*(159) = 2.91, *p* = 0.033, *d* = 0.45) phenotype. Participants with the SA phenotype showed lower levels of hyperactivity than those with all other phenotypes (GSD: *t*(162) =  − 6.57, *p* < 0.001, *d* = 0.86; TSS: *t*(150) =  − 4.75, *p* < 0.001, *d* = 0.70; URSS: *t*(197) =  − 6.19, *p* < 0.001, *d* = 0.85; M/LEW: *t*(169) =  − 4.18, *p* < 0.001, *d* = 0.60). In addition, the internal consistency of the SWAN was assessed, with Cronbach's *α* = 0.921, indicating excellent internal consistency.

### OCD traits

TOCS scores indicated that OCD traits differed significantly across the sensory phenotypes (*F*(4, 196.3) = 5.21, *p* < 0.001, est. *w*^2^ = 0.039; see Fig. 6 and Table 2). Post hoc comparisons indicated participants with the M/LEW phenotype had more OCD traits than those with the SA phenotype (*t*(174) = 2.81, *p* = 0.043, *d* = 0.42), while those with the GSD phenotype had more OCD traits than those with the SA (*t*(159) = 3.71, *p* = 0.003, *d* = 0.56) and URSS (*t*(151) = 3.42, *p* = 0.007, *d* = 0.55) phenotypes. In addition, the internal consistency of the TOCS was assessed, with Cronbach's *α* = 0.946, indicating excellent internal consistency.

## Discussion

The aim of this study was to examine patterns of sensory processing in autistic individuals in order to identify and describe potential sensory phenotypes. To this end, we were able to describe five sensory phenotypes. Further, differences in age, adaptive behaviour, and traits associated with autism, attention-deficit and hyperactivity disorder, and obsessive and compulsive disorder, differed across the five sensory phenotypes.

Much like previous studies that have attempted to cluster sensory behaviours in ASD, the first two phenotypes to emerge from our data were a *sensory adaptive (SA)* phenotype and a *generalized sensory difference (GSD)* phenotype. Individuals characterized by the SA phenotype were reported by their parents to have typical sensory performance across tactile, taste/smell, movement, under-responsive/sensory seeking, and visual/ auditory processing, although they did show probable differences on the auditory filtering and low energy/weakness subscales of the SSP relative to normative data from a non-clinical sample [10]. On the other hand, individuals characterized by the GSD phenotype were reported by their parents to have definite differences across all SSP subscales. Having both a SA and a GSD phenotype suggests that our phenotypes span the full spectrum of sensory abilities [4]. We also identified three additional sensory phenotypes that demonstrated typical performance on select subscales while exhibiting definite differences on others.

In addition to the SA and GSD phenotypes, a *taste and smell sensitivity (TSS)* phenotype was identified that characterized individuals reported to have typical performance on the movement and low energy/weakness subscales, with definite differences on the remaining five subscales. Notably, this group showed particularly high taste and smell sensitivity. The *under-responsive/sensory seeking (URSS)* phenotype characterized individuals who were reported to have definite differences in under responsivity and sensory seeking, as well as auditory filtering difficulties, probable differences in visual/auditory and tactile processing, and typical performance with regards to taste/smell, movement, and low energy/weakness. Lastly, individuals characterized by the *movement difficulties and low energy/weak (M/LEW)* phenotype were reported to have definite differences in movement, under-responsiveness and sensory seeking, auditory filtering, and low energy and weakness, with probable differences in tactile, taste and smell, and visual and auditory processing. The TSS [4–6], URSS [5], and M/LEW [6] phenotypes have been previously described in other sensory clustering attempts, but inconsistently, likely due to smaller sample sizes.

The five sensory phenotypes identified were associated with demographic and behavioural traits commonly observed in autistic individuals. Similar to previous sensory clustering studies, the autistic individuals who demonstrated the most sensory processing differences (here the GSD phenotype) also had the lowest adaptive functioning scores [4, 14, 47], and the most restricted and repetitive behaviours [2, 51], social communication difficulties [14, 51], and hyperactive, inattentive, and compulsive behaviours [14]. Likewise, the most adaptive sensory processing subtype (here the SA phenotype) also showed the highest levels of adaptive behaviours [14, 47], with relatively low levels of repetitive behaviours [14, 47], social communication difficulties [14, 47], and hyperactive, inattentive, and compulsive behaviours [14]. Despite being on the opposite extremes of the sensory processing spectrum, individuals in the SA and GSD phenotypes did not differ in age, IQ, or sex at birth, in line with previous sensory clustering studies [4, 5, 51], Importantly, this suggests that while individuals characterized by the GSD phenotype have the most sensory processing difficulties, these individuals are not more cognitively impaired. It should be noted here that 17.2% of the current population had an IQ below 70, suggesting that this lack of difference in cognitive ability was not due to including only autistic individuals with higher IQs, though this is below the 33% estimated in the population [52].

Considering the intermediate phenotypes, or the TSS, URSS, and M/LEW phenotypes, individuals characterized by the URSS and M/LEW phenotypes showed poor adaptive functioning, suggesting that autistic individuals who demonstrate particularly high levels of underresponsivity and sensory seeking, movement difficulties, and low energy, may be those who will benefit most from environments that support these sensory differences (e.g. heightened sensory environments with increased physical accessibility modifications). On the other hand, individuals characterized by the TSS phenotype scored high on repetitive behaviours and hyperactivity, suggesting autistic individuals with taste and smell difficulties may respond well to, and benefit the most from, environments with supports aiming to help better manage repetitive behaviours and hyperactivity. Individuals characterized by the TSS phenotype were also the youngest, providing support for previous work that has found that repetitive behaviours become less severe with age [53]. Together these findings suggest that sensory processing difficulties are strongly related to other behavioural difficulties commonly observed in autistic individuals, thus aiming to modify environments in such a manner that sensory difficulties are not further exacerbated may serve to reduce other behavioural difficulties. Further, these results suggest that these phenotypes may not only be beneficial for parsing sensory processing heterogeneity, but autism traits and traits more broadly. Importantly, grouping autistic individuals by these phenotypes could allow for more focused interventions that target the sensory domains, and behaviours, that present the most difficulty for those individuals [4–6, 54, 55]. For example, better understanding autistic children’s sensory abilities could help teachers modify classrooms in such a manner that capitalize on the child’s sensory abilities, while also supporting their sensory difficulties. Notably, these results also suggest that there is a subset of autistic individuals with relatively less sensory processing difficulties. Although participants who were characterized by the SA phenotype also showed fewer behavioural difficulties, taken together these findings suggest that sensory-based interventions may be less effective, or even less necessary, for this group of autistic individuals [11].

Classification of sensory processing differences into sensory phenotypes also has practical significance for researchers interested in behaviours that are impacted by sensory processing. If autistic individuals are treated as a homogenous group when measuring a behaviour that is influenced by sensory processing, the heterogeneity in the autistic individuals’ sensory behaviours may mask differences in the behaviour of interest. By classifying autistic individuals by their sensory phenotype, researchers will have a better chance of accurately identifying differences in their behaviour of interest.

While sensory processing difficulties are common for autistic individuals, these difficulties are also seen in other developmental disorders such as ADHD [56, 57] and OCD [58, 59], as well as in neurodevelopmentally typical children [60] and adults [61, 62]. Future work should investigate whether the sensory phenotypes identified here are autism-specific, or can also be observed in other populations. It is also important to investigate the stability of these sensory phenotypes over time, as the current study identified age differences across the sensory phenotypes. Further, given the utility of clustering techniques for parsing the heterogeneity in traits and behaviours that has already been demonstrated [63–65], future work should apply these clustering techniques to traits such as anxiety, restricted and repetitive behaviours, and social communication difficulties to determine whether discrete phenotypes can also be identified. In addition, while these sensory phenotypes were related to current behavioural differences, future work should aim to determine whether these phenotypes are also predictive of future behaviours. Given the sensory phenotypes identified here were related to autism traits, if phenotypes related to other autistic traits can be identified, it may be possible to map these phenotypes onto one another to create more detailed clinical profiles within the ASD diagnosis. Further refining the ASD diagnosis by identifying and describing specific clinical profiles will assist in producing interventions tailored to support, rather than create barriers to, the needs of the individual. In addition, reducing heterogeneity may aid in the identification of specific genetic profiles, as well as outcome profiles. With that in mind, future work must also investigate whether these sensory profiles moderate intervention responses.

## Limitations

It is important to acknowledge the limitations of this study. Given the results were based on subjective parent-report measures of sensory processing, adaptive behaviour, and traits associated with autism, attention-deficit and hyperactivity disorder, and obsessive and compulsive disorder, this limits the generalizability of the findings. It is also important to note that the current sample spanned a large age range, and many of the measures used produced unstandardized scores. To ensure the age range did not confound the current results, we ran exploratory analyses of covariance for the variables that were significantly correlated with age to ensure that the results were not misrepresented by the Welch’s ANOVAs reported here (see Additional file 3). Further, while the SSP is widely used to measure sensory processing in autistic children and adults, there is limited psychometric evidence of convergent validity [66]. Future work will aim to replicate and refine these findings with the use of alternate sensory questionnaires (e.g. Sensory Profile 2 [67], Sensory Experiences Questionnaire 3 [68], as well as behavioural, neural, and genetic methodologies. In the current sample, 17.2% of the individuals had an intellectual disability (ID; IQ less than 70), while estimates of ID in the autistic population are currently ~ 33% [52], suggesting we underrepresented autistic individuals with ID in our sample. Further, as these data were obtained from established records from a large provincial database, not all measures were completed for all individuals.

## Conclusion

These findings suggest that sensory difficulties in autistic individuals can be clustered into sensory phenotypes that parset some of the heterogeneity in sensory issues in autism. These discrete sensory phenotypes are associated with unique behavioural/clinical profiles. Given that these sensory phenotypes do not differ in IQ or sex ratio, these results do not appear to be the result of differences in cognitive ability or sex assigned at birth. Thus, these results suggest that sensory issues may provide a novel way to understand behavioural heterogeneity in autism.

## Supplementary Information

**Additional file 1.** Bayesian Information Criteria (BIC) values for k-means 2-6 models are plotted. Further, the results of a bootstrapping procedure that produced 100 iterations of the 5 cluster solution is presented.

**Additional file 2.** Full statistics on the one-way ANOVAs conducted to determine whether SSP subscale scores differed across the 5 sensory phenotypes are presented. Further, Cronbach's Alpha values for each subscale, as well as the total SSP score are presented.

**Additional file 3.** Correlations between all experimental variables are presented. Results of the analyses of covariances (ANCOVAs) for variables that correlated significantly with age are also presented for comparison.

**Additional file 4.** Full statistics on the one-way ANOVAs conducted to determine whether the VABS-II Adaptive Behaviour subscales differ across the 5 sensory phenotypes are presented.

**Additional file 5.** Full statistics on the one-way ANOVAs conducted to determine whether the RBS-R Repetitive Beahviours subscales differ across the 5 sensory phenotypes are presented.

## Data Availability

The data that support the findings of this study are available from POND, but restrictions apply to the availability of these data, which were used under licence for the current study, and so are not publicly available. Data are however available from the authors upon reasonable request and with permission of POND.

## Abbreviations

ADHD: Attention-deficit/hyperactivity disorder
ADI-R: Autism diagnostic interview
ADOS: Autism diagnostic observation schedule
ANOVA: Analysis of variance
APA: American Psychological Association
ASD: Autism spectrum disorder
BIC: Bayesian information criterion
DSM: Diagnostic and statistical manual of mental disorders
GSD: Generalized sensory difference
ID: Intellectual disability
IQ: Intelligence quotient
M: Mean
M/LEW: Movement with low energy and weakness
OCD: Obsessive compulsive disorder
POND: Province of Ontario neurodevelopmental disorders network
RRBs: Restricted and repetitive behaviours
RBS-R: Repetitive Behaviours Scale—Revised
SA: Sensory adaptive
SCQ: Social Communication Questionnaire
SD: Standard deviation
SSP: Short sensory profile
SPSS: Statistical package for the social sciences
SWAN: Strengths and Weaknesses of Attention-Deficit/Hyperactivity Disorder Symptoms of Normal Behaviour Scale
TOCS: Toronto Obsessive–Compulsive Scale
TSS: Taste and smell sensitivity
URSS: Under-responsive and sensory seeking
VABS-II: Vineland Adaptive Behaviour Scales—Second Edition
