# Supplementary material for: Exploring sensory phenotypes in autism spectrum disorder
Source: Mol Autism. 2021 Oct 12;12:67. doi: 10.1186/s13229-021-00471-5 (PMC8507349; doi:10.1186/s13229-021-00471-5)
Supplement: Supplementary file 1 — Additional file 1. Bayesian Information Criteria (BIC) values for k-means 2-6 models are plotted. Further, the results of a bootstrapping procedure that produced 100 iterations of the 5 cluster solution is presented. [file 13229_2021_471_MOESM1_ESM.docx]

Supplemental Materials A

Bayesian information criteria (BIC) was used to assess the fit of the model. Similar reductions in variance were observed for the 5 and 6 cluster solutions (see Figure 1).

Figure 1: Bayesian Information Criteria (BIC) values for K-means 2-6 models.

To determine the reliability of the final clustering solution, a bootstrapping procedure was used to generate 100 iterations of the five-cluster solution. The mean of the 100 iterations closely resembled the adopted solution, while the standard deviation of the 100 iterations indicated the variance across the iterations was low (see Figure 2). Specifically, the standard deviation of each domain score across all 5 sensory phenotypes was <.01, indicating negligible variance in these cluster solutions across the 100 iterations.


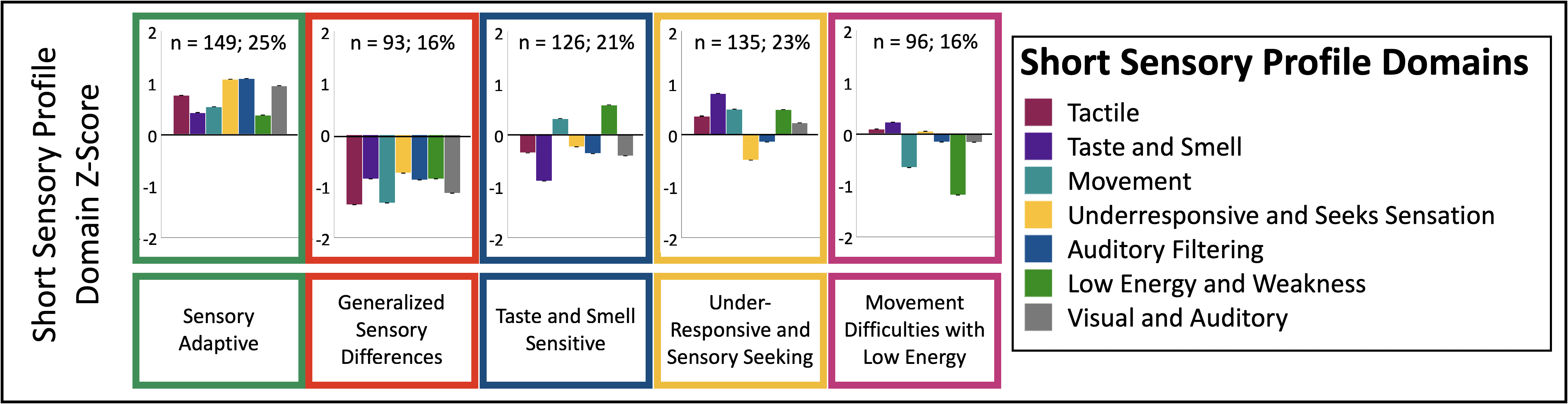


Figure 2: Mean and standard deviation of 100 iterations of the 5-cluster solution.
