# Supplementary material for: Exploring sensory phenotypes in autism spectrum disorder
Source: Mol Autism. 2021 Oct 12;12:67. doi: 10.1186/s13229-021-00471-5 (PMC8507349; doi:10.1186/s13229-021-00471-5)
Supplement: Supplementary file 2 — Additional file 2. Full statistics on the one-way ANOVAs conducted to determine whether SSP subscale scores differed across the 5 sensory phenotypes are presented. Further, Cronbach's Alpha values for each subscale, as well as the total SSP score are presented. [file 13229_2021_471_MOESM2_ESM.docx]

Supplemental Materials B

Table 1: One way ANOVAs were conducted on the Short Sensory Profile (SSP) subscale scores to determine whether SSP subscales differed across the 5 sensory phenotypes (SA – Sensory Adaptive, GSD – Generalized Sensory Differences, TSS – Taste and Smell Sensitivity, URSS – Underresponsive and Sensory Seeking), and M/LEW – Movement and Low Energy / Weakness).

| SSP Subscale | ANOVA | Games-Howell Post-Hoc |  |  |  |  |
| --- | --- | --- | --- | --- | --- | --- |
|  |  | Sensory Phenotype | GSD | TSS | URSS | M/LEW |
| Tactile | F(4, 280.2) = 135.35, p < .001, est. 𝑤^2^ = .473. | SA | t(185) = 21.9, p < .001, *d* = 2.91 | t(250) = 12.12, p < .001, *d* = 1.47 | t(282) = 4.70, p < .001, *d* = .56 | t(210) = 7.42, p < .001, *d* = .97 |
|  |  | GSD | - | t(206) = -9.44, p < .001, *d* = 1.28 | t(179) = -18.11, p < .001, *d* = 2.47 | t(183) = -14.07, p < .001, *d* = 2.05 |
|  |  | TSS | - | - | t(24-) = -8.10, p < .001, *d* = 1.01 | t(219) = -4.60, p < .001, *d* = .62 |
|  |  | URSS | - | - | - | t(202) = 3.21, p = .013, *d* = .43 |
|  |  |  |  |  |  |  |
|  |  |  | GSD | TSS | URSS | M/LEW |
| Taste/Smell | F(4, 274.2) = 193.24, p < .001, est. 𝑤^2^ = .562. | SA | t(226) = 12.8, p < .001, *d* = 1.65 | t(269) = 14.46, p < .001, *d* = 1.73 | t(240) = -4.67,  p < .001, *d* = .55 | t(198) = 1.7-, p = .434, *d* = .22 |
|  |  | GSD | - | t(192) = .370, p = .996, *d* = .05 | t(156) = -20.11, p < .001, *d* = 2.79 | t(178) = -9.44, p < .001, *d* = 1.37 |
|  |  | TSS | - | - | t(234) = -23.76, p < .001, *d* = 2.96 | t(167) = -10.45, p < .001, *d* = 1.45 |
|  |  | URSS | - | - | - | t(137) = 5.80, p < .001, *d* = .81 |
|  |  |  |  |  |  |  |
|  |  |  |  |  |  |  |
|  |  |  | GSD | TSS | URSS | M/LEW |
| Movement | F(4, 268.0) = 105.109, p < .001, est. 𝑤^2^ = .410. | SA | t(134) = 17.8, p < .001, *d* = 2.47 | t(257) = 3.21, p = .013, *d* = .39 | t(270) = .60, p = .975, *d* = .07 | t(135) = 10.99, p < .001, *d* = 1.52 |
|  |  | GSD | - | t(148) = -15.11, p < .001, *d* = 2.13 | t(149) = -16.76, p < .001, *d* = 2.34 | t(187) = -4.92, p < .001, *d* = .72 |
|  |  | TSS | - | - | t(259) = -2.41, p = .115, *d* = .30 | t(148) = 8.65, p < .001, *d* = 1.21 |
|  |  | URSS | - | - | - | t(149) = 10.26, p < .001, *d* = 1.42 |
|  |  |  |  |  |  |  |
|  |  |  | GSD | TSS | URSS | M/LEW |
| Underresponsive/ Sensory Seeking | F(4, 276.1) = 135.52, p < .001, est. 𝑤^2^ = .473. | SA | t(161) = 17.9, p < .001, *d* = 2.42 | t(238) = 14.53, p < .001, *d* = 1.77 | t(276) = 19.53, p < .001, *d* = 2.33 | t(181) = 10.87, p < .001, *d* = 1.45 |
|  |  | GSD | - | t(196) = -4.48, p < .001, *d* = .62 | t(172) = -2.47, p = .102, *d* = .34 | t(184) = -6.83, p < .001, *d* = 1.00 |
|  |  | TSS | - | - | t(245) = 2.64, p = .066, *d* = .32 | t(211) = -2.70, p = .058, *d* = .36 |
|  |  | URSS | - | - | - | t(192) = -5.47, p < .001, *d* = .74 |
|  |  |  |  |  |  |  |
|  |  |  |  |  |  |  |
|  |  |  |  |  |  |  |
|  |  |  | GSD | TSS | URSS | M/LEW |
| Auditory Filtering | F(4, 283.2) = 130.72, p < .001, est. 𝑤^2^ = .464. | SA | t(219) = 21.80, p < .001, *d* = 2.83 | t(267) = 16.18, p < .001, *d* = 1.96 | t(278) = 13.69, p < .001, *d* = 1.63 | t(198) = 12.43, p < .001, *d* = 1.63 |
|  |  | GSD | - | t(212) = -5.48, p < .001, *d* = .74 | t(218) = -7.89, p < .001, *d* = 1.05 | t(182) = -6.97, p < .001, *d* = 1.01 |
|  |  | TSS | - | - | t(259) = -2.42, p = .113, *d* = .30 | t(199) = -2.03, p = .255, *d* = .27 |
|  |  | URSS | - | - | - | t(202) = .15, p = 1.000, *d* = .02 |
|  |  |  |  |  |  |  |
|  |  |  | GSD | TSS | URSS | M/LEW |
| Low Energy / Weak | F(4, 276.2) = 155.25, p < .001, est. 𝑤^2^ = .507. | SA | t(177) = 10.7, p < .001, *d* = 1.43 | t(262) = -2.42, p = .112, *d* = .29 | t(271) = -1.43, p = .606, *d* = .17 | t(228) = 16.41, p < .001, *d* = 2.10 |
|  |  | GSD | - | t(140) = -13.42, p < .001, *d* = 1.90 | t(145) = -12.56, p < .001, *d* = 1.76 | t(168) = 2.80, p = .045, *d* = .41 |
|  |  | TSS | - | - | t(259) = 1.13, p = .792, *d* = .14 | t(181) = 20.92, p < .001, *d* = 2.87 |
|  |  | URSS | - | - | - | t(189) = 19.69, p < .001, *d* = 2.66 |
|  |  |  |  |  |  |  |
|  |  |  |  |  |  |  |
|  |  |  |  |  |  |  |
|  |  |  |  |  |  |  |
|  |  |  |  |  |  |  |
|  |  |  | GSD | TSS | URSS | M/LEW |
| Visual / Auditory | F(4, 273.7) = 174.30, p < .001, est. 𝑤^2^ = .547. | SA | t(162) = 24.4, p < .001, *d* = 3.31 | t(214) = 15.99, p < .001, *d* = 1.97 | t(245) = 9.04, p < .001, *d* = 1.08 | t(150) = 11.66, p < .001, *d* = 1.59 |
|  |  | GSD | - | t(212) = -7.06, p < .001, *d* = .96 | t(206) = -14.18, p < .001, *d* = 1.90 | t(184) = -8.79, p <.001, *d* = 1.28 |
|  |  | TSS | - | - | t(253) = -6.75, p < .001, *d* = .84 | t(203) = -2.28, p = .157, *d* = .31 |
|  |  | URSS | - | - | - | t(192) = 3.79, p = .002, *d* = .51 |
|  |  |  |  |  |  |  |
|  |  |  |  |  |  |  |

Table 2: Cronbach’s Alpha was calculated for the SSP and it’s subscales.

| Scale | Cronbach’s Alpha |
| --- | --- |
| Tactile | .775 |
| Taste and Smell | .919 |
| Movement | .786 |
| Under Responsive Sensory Seeking | .847 |
| Auditory Filtering | .830 |
| Low Energy Weakness | .932 |
| Visual Auditory | .837 |
| Total SSP Score | .917 |
