# Supplementary material for: Exploring sensory phenotypes in autism spectrum disorder
Source: Mol Autism. 2021 Oct 12;12:67. doi: 10.1186/s13229-021-00471-5 (PMC8507349; doi:10.1186/s13229-021-00471-5)
Supplement: Supplementary file 3 — Additional file 3. Correlations between all experimental variables are presented. Results of the analyses of covariances (ANCOVAs) for variables that correlated significantly with age are also presented for comparison. [file 13229_2021_471_MOESM3_ESM.docx]

| **Supplemental Materials C**  *Pearson Bivariate Correlations Between Main Variables* | | | | | | | | | | | | | | | | | | | | | | | | | |
| --- | --- | --- | --- | --- | --- | --- | --- | --- | --- | --- | --- | --- | --- | --- | --- | --- | --- | --- | --- | --- | --- | --- | --- | --- | --- |
|  | 1 | 2 | 3 | 4 | 5 | 6 | 7 | 8 | 9 | 10 | 11 | 12 | 13 | 14 | 15 | 16 | 17 | 18 | 19 | 20 | 21 | 22 | 23 | 24 | 25 |
| 1. SSP - Tactile |  |  |  |  |  |  |  |  |  |  |  |  |  |  |  |  |  |  |  |  |  |  |  |  |  |
| 1. SSP – Taste and Smell | 0.384^**^ |  |  |  |  |  |  |  |  |  |  |  |  |  |  |  |  |  |  |  |  |  |  |  |  |
| 1. SSP - Movement | 0.414^**^ | 0.247^**^ |  |  |  |  |  |  |  |  |  |  |  |  |  |  |  |  |  |  |  |  |  |  |  |
| 1. SSP – Underresponsive and Sensory Seeking | 0.346^**^ | 0.173^**^ | 0.163^**^ |  |  |  |  |  |  |  |  |  |  |  |  |  |  |  |  |  |  |  |  |  |  |
| 1. SSP – Auditory Filtering | 0.463^**^ | 0.266^**^ | 0.253^**^ | 0.517^**^ |  |  |  |  |  |  |  |  |  |  |  |  |  |  |  |  |  |  |  |  |  |
| 1. SSP – Low Energy Weak | 0.227^**^ | 0.099* | 0.446^**^ | 0.117^*^ | 0.197^**^ |  |  |  |  |  |  |  |  |  |  |  |  |  |  |  |  |  |  |  |  |
| 1. SSP – Visual Auditory | 0.557^**^ | 0.369^**^ | 0.413^**^ | 0.400^**^ | 0.527^**^ | 0.220^**^ |  |  |  |  |  |  |  |  |  |  |  |  |  |  |  |  |  |  |  |
| 1. Age | 0.000 | 0.088^*^ | -0.127^*^ | 0.204^**^ | 0.044 | -0.281^**^ | 0.039 |  |  |  |  |  |  |  |  |  |  |  |  |  |  |  |  |  |  |
| 1. Full IQ | -0.112^*^ | -0.004 | 0.037 | 0.126^*^ | -0.142^**^ | -0.101^*^ | -0.022 | 0.112^*^ |  |  |  |  |  |  |  |  |  |  |  |  |  |  |  |  |  |
| 1. Verbal IQ | -0.115^*^ | -0.023 | 0.013 | 0.106^*^ | -0.122^*^ | -0.096^*^ | 0.008 | -0.035 | 0.945^**^ |  |  |  |  |  |  |  |  |  |  |  |  |  |  |  |  |
| 1. Perform IQ | -0.058 | -0.017 | 0.093^*^ | 0.127^*^ | -0.118^*^ | -0.023 | 0.019 | -0.052 | 0.936^**^ | 0.788^**^ |  |  |  |  |  |  |  |  |  |  |  |  |  |  |  |
| 1. VABS – Adaptive Behaviour Composite Score | 0.085 | 0.106^*^ | 0.100^*^ | 0.250^**^ | 0.088 | 0.141^*^ | 0.087 | -0.307^**^ | 0.532^**^ | 0.518^**^ | 0.556^**^ |  |  |  |  |  |  |  |  |  |  |  |  |  |  |
| 1. VABS – Communication Skills | 0.046 | 0.079 | 0.058 | 0.223^**^ | 0.087 | 0.064 | 0.042 | -0.253^**^ | 0.554^**^ | 0.557^**^ | 0.560^**^ | 0.913^**^ |  |  |  |  |  |  |  |  |  |  |  |  |  |
| 1. VABS – Daily Living Skills | 0.101^*^ | 0.114^*^ | 0.129^*^ | 0.229^**^ | 0.070 | 0.164^**^ | 0.093 | -0.265^**^ | 0.523^**^ | 0.488^**^ | 0.546^**^ | 0.931^**^ | 0.820^**^ |  |  |  |  |  |  |  |  |  |  |  |  |
| 1. VABS – Socialization Skills | 0.126^*^ | 0.105^*^ | 0.069 | 0.262^**^ | 0.102^*^ | 0.108^*^ | 0.092 | -0.251^**^ | 0.387^**^ | 0.355^**^ | 0.422^**^ | 0.892^**^ | 0.728^**^ | 0.772^**^ |  |  |  |  |  |  |  |  |  |  |  |
| 1. VABS – Motor Skills | -0.093 | 0.155 | 0.234^*^ | 0.192^*^ | 0.059 | 0.205^*^ | -0.040 | -0.158 | 0.534^*^ | 0.524^**^ | 0.603^**^ | 0.837^**^ | 0.614^**^ | 0.744^**^ | 0.648^**^ |  |  |  |  |  |  |  |  |  |  |
| 1. RBS-R - Total | -0.500^**^ | -0.384^**^ | -0.277^**^ | -0.452^**^ | -0.404^**^ | -0.122^*^ | -0.519^**^ | -0.048 | -0.122^*^ | -0.133^*^ | -0.121^*^ | -0.155^*^ | -0.096^*^ | -0.149^*^ | -0.226^**^ | -0.004 |  |  |  |  |  |  |  |  |  |
| 1. RBS-R – Self-Injury | -0.258^**^ | -0.212^**^ | -0.139^**^ | -0.319^**^ | -0.213^**^ | -0.055 | -0.294^**^ | 0.017 | -0.169^**^ | -0.181^**^ | -0.199^**^ | -0.250^**^ | -0.219^**^ | -0.217^**^ | -0.251^**^ | -0.083 | 0.588^**^ |  |  |  |  |  |  |  |  |
| 1. RBS-R – Stereotype | -0.302^**^ | -0.269^**^ | -0.153^**^ | -0.539^**^ | -0.313^**^ | 0.017 | -0.401^**^ | -0.203^**^ | -0.232^**^ | -0.194^**^ | -0.208^**^ | -0.225^**^ | -0.186^**^ | -0.223^**^ | -0.267^**^ | -0.141 | 0.744^**^ | 0.449^**^ |  |  |  |  |  |  |  |
| 1. RBS-R – Ritualistic/ Sameness | -0.513^**^ | -0.405^**^ | -0.301^**^ | -0.361^**^ | -0.394^**^ | -0.165^**^ | -0.495^**^ | 0.007 | -0.024 | -0.057 | -0.014 | -0.071 | -0.007 | -0.072 | -0.155^**^ | 0.078 | 0.915^**^ | 0.376^**^ | 0.514^**^ |  |  |  |  |  |  |
| 1. RBS-R – Compulsion | -0.352^**^ | -0.256^**^ | -0.175^**^ | -0.299^**^ | -0.273^**^ | -0.058 | -0.367^**^ | -0.083^*^ | -0.159^**^ | -0.157^**^ | -0.157^**^ | -0.081 | -0.041 | -0.094 | -0.132^*^ | -0.007 | 0.836^**^ | 0.337^**^ | 0.596^**^ | 0.702^**^ |  |  |  |  |  |
| 1. SCQ | -0.224^**^ | -0.163^**^ | -0.152^**^ | -0.362^**^ | -0.238^**^ | -0.128^*^ | -0.293** | 0.068 | -0.320^**^ | -0.322^**^ | -0.310^**^ | -0.466^**^ | -0.389^**^ | -0.449^**^ | -0.468^**^ | -0.354^**^ | 0.379^**^ | 0.286^**^ | 0.379^**^ | 0.308^**^ | 0.277^**^ |  |  |  |  |
| 1. SWAN I | -0.116^*^ | -0.028 | -0.093^*^ | -0.401^**^ | -0.368^**^ | -0.118^*^ | -0.122^*^ | -0.026 | -0.251^**^ | -0.229^**^ | -0.265^**^ | -0.494^**^ | -0.482^**^ | -0.470^**^ | -0.410^**^ | -0.469^*^ | 0.183^**^ | 0.230^**^ | 0.248^**^ | 0.132^*^ | 0.049 | 0.294^**^ |  |  |  |
| 1. SWAN HI | -0.142^*^ | -0.100 | -0.037 | -0.545^**^ | -0.339^**^ | -0.006 | -0.158^**^ | -0.211^**^ | -0.209^**^ | -0.178^**^ | -0.233^**^ | -0.341^**^ | -0.303^**^ | -0.312^**^ | -0.351^**^ | -0.263^**^ | 0.314^**^ | 0.276^**^ | 0.405^**^ | 0.230^**^ | 0.190^**^ | 0.310^**^ | 0.640^**^ |  |  |
| 1. TOCS | -0.253^**^ | -0.128^*^ | -0.150^*^ | 0.016 | -0.227^**^ | -0.135^*^ | -0.190^**^ | -0.007 | 0.210^**^ | 0.243^**^ | 0.214^**^ | 0.222^**^ | 0.227^**^ | 0.194^**^ | 0.204^**^ | -0.014 | 0.242^**^ | -0.030 | 0.057 | 0.265^**^ | 0.323^**^ | 0.028 | -0.137^*^ | -0.121^*^ |  |

Abbreviation: SSP, Short Sensory Profile; IQ, Intelligence Quotient; VABS-II, Vineland Adaptive Behavioral Scales; RBS-R, – Repetitive Behaviour Scale – Revised; SCQ, Social Communication Questionnaire; TOCS, Toronto Obsessive-Compulsive Scale; SWAN I, Strengths and Weaknesses of Attention-Deficit/Hyperactivity Disorder Symptoms of Normal Behavior Scale Inattentive Scale; SWAN HI, Strengths and Weaknesses of Attention-Deficit/Hyperactivity Disorder Symptoms of Normal Behavior Scale Hyperactivity Scale

Note. * *p* < .05, ** *p* ≤ .001,

Given the significant correlation between age and some of the unstandardized measures, we ran exploratory analyses of covariance (ANCOVAs) for the variables that were significantly correlated with age to ensure that we were not misrepresenting our results by using Welch’s ANVOAs (where we cannot control for a covariate). Below on the left you will see the statistics as currently reported, and on the right you will see the F-statistics for the measures that result from the ANCOVA. In about half of the cases, age was a significant covariate. Despite that, adding age as a covariate did not change the outcome of the measure’s F-statistic, nor did it effect the posthoc comparisons. For this reason, we are confident that we are not misrepresenting the findings by not including age as a covariate.

Taste and Smell: **Age not a significant covariate (p = .515)

No Covariate: F (4, 274) = 193.2, p < .001 With Covariate F(4, 593) = 134.92, p < .001

SA different than GSD, TSS, URSS, *All posthocs remained the same

GSD & TSS different than URSS, M/LEW

URSS different than M/LEW

Movement **Age was not a significant covariate (p = .181)

No Covariate F (4, 268) = 105.1, p < .001 With Covariate F(4, 593) = 138.55, p < .001

SA different than GSD, TSS, M/LEW *All posthocs remained the same

GSD different than TSS, URSS, M/LEW

TSS & URSS different than M/LEW

Underresponsive Sensory Seeking **Age was a significant covariate (p < .001)

No Covariate: F (4, 276) = 135.5, p < .001 With Covariate F(4, 593) = 116, p < .001

SA different than GSD, TSS, URSS, M/LEW *All posthocs remained the same

GSD different than TSS, M/LEW

URSS different than M/LEW

Low Energy Weakness **Age was a significant covariate (p < .001)

No Covariate: F (4, 276) = 155.2, p < .001 With Covariate F(4, 593) = 136.4, p < .001

SA different than GSD, M/LEW *All posthocs remained the same

GSD different than TSS, URSS, M/LEW

TSS and URSS different than M/LEW

RBS-R Stereotypy **Age was a significant covariate (p < .001)

No Covariate: F (4, 259) = 43.8, p < .001 With Covariate F(4, 561) = 34.8, p < .001

SA different than GSD, TSS, URSS, M/LEW *All posthocs remained the same

GSD and TSS different than URSS, M/LEW

RBS-R Compulsions **Age was not a significant covariate (p = .078)

No Covariate: F (4, 252) = 25.8, p < .001 With Covariate F(4, 561) = 24.68, p < .001

SA different than GSD, TSS, URSS, M/LEW *All posthocs remained the same

GSD different than URSS, M/LEW

TSS different than URSS

SWAN Hyperactivity **Age was a significant covariate (p < .001)

No Covariate: F (4, 221) = 15.5, p < .001 With Covariate F(4, 459) = 11.2, p < .001

SA different than GSD, TSS, URSS, M/LEW *All posthocs remained the same

VABS-II Adaptive Behaviour Composite **Age was a significant covariate (p < .001)

*F*(4, 203.5) = 8.62, *p* < .001 With Covariate F(4, 428) = 8.727, p < .001

SA > GSD, URSS, M/LEW; GSD < TSS SA > GSD, TSS, URSS, M/LEW, GSD < TSS (p = .06)

VABS-II Communication Skills **Age was a significant covariate (p < .001)

*F*(4, 209.0) = 5.58, *p* < .001 With Covariate F(4, 435) = 6.557, p < .001

SA > GSD, URSS, M/LEW SA > GSD, TSS, URSS, M/LEW,

VABS-II Daily Living Skills **Age was a significant covariate (p < .001)

*F*(4, 207.3) = 9.51, *p* < .001 With Covariate F(4, 434) = 8.398, p < .001

GSD < SA, TSS, URSS; M/LEW < SA, TSS GSD < SA, TSS, URSS; M/LEW. URSS, TSS < SA

VABS-II Socialization Skills **Age was a significant covariate (p < .001)

*F*(4, 207.9) = 7.40, *p* < .001 With Covariate F(4, 434) = 8.020, p < .001

SA > GSD, URSS, M/LEW; TSS > GSD SA > GSD, TSS, URSS, M/LEW; TSS > GSD (p = .06)

Note: SA = Sensory Adaptive, GSD = Generalized Sensory Difference, TSS = Taste and Smell Sensitivity, URSS = Underresponsive / Seeks Sensation, M/LEW = Movement with Low Energy and Weakness.
