# Supplementary material for: Exploring sensory phenotypes in autism spectrum disorder
Source: Mol Autism. 2021 Oct 12;12:67. doi: 10.1186/s13229-021-00471-5 (PMC8507349; doi:10.1186/s13229-021-00471-5)
Supplement: Supplementary file 4 — Additional file 4. Full statistics on the one-way ANOVAs conducted to determine whether the VABS-II Adaptive Behaviour subscales differ across the 5 sensory phenotypes are presented. [file 13229_2021_471_MOESM4_ESM.docx]

Supplemental Materials D - Welch’s One way ANOVAs were conducted on the Vineland Adaptive Behaviour Scales – Second Edition (VABS) scores to determine whether VABS-II subscales differed across the 5 sensory phenotypes (SA – Sensory Adaptive, GSD – Generalized Sensory Differences, TSS – Taste and Smell Sensitivity, URSS – Underresponsive and Sensory Seeking), and M/LEW – Movement and Low Energy / Weakness).

| VABS Subscale | Welch’s ANOVA | Games-Howell Post-Hoc |  |  |  |  |
| --- | --- | --- | --- | --- | --- | --- |
|  |  | Sensory Phenotype | GSD | TSS | URSS | M/LEW |
| Adaptive Behaviour Composite | F(4, 203.5) = 8.62, p < .001, est. 𝑤^2^ = .066. | SA | t(169) = 5.34, p < .001, *d* = .80 | t(188) = 1.94, p = .298, *d* = .28 | t(193) = 2.90, p = .033, *d* = .41 | t(139) = 4.03, p < .001, *d* = .63 |
|  |  | GSD | - | t(152) = -3.57, p = .004, *d* = .57 | t(167) = -2.09, p = .228, *d* = .32 | t(130) = -.65, p = .966, *d* = .11 |
|  |  | TSS | - | - | t(176) = 1.18, p = .764, *d* = .17 | t(128) = 2.44, p = .110, *d* = .40 |
|  |  | URSS | - | - | - | t(149) = 1.25, p = .725, *d* = .20 |
|  |  |  |  |  |  |  |
|  |  |  | GSD | TSS | URSS | M/LEW |
| Communication Skills | F(4, 209.0) = 5.58, p < .001, est. 𝑤^2^ = .040. | SA | t(173) = 4.11, p < .001, *d* = .61 | t(186) = 2.23, p = .173, *d* = .32 | t(194) = 3.13,  p = .017, *d* = .44 | t(152) = 3.76, p = .002, *d* = .58 |
|  |  | GSD | - | t(159) = -1.79, p = .381, *d* = .28 | t(170) = -.68, p = .960, *d* = .10 | t(138) = .02, p = 1.000, *d* = .00 |
|  |  | TSS | - | - | t(181) = 1.00, p = .856, *d* = .15 | t(147) = 1.66, p = .461, *d* = .27 |
|  |  | URSS | - | - | - | t(160) = .65, p = .966, *d* = .10 |
|  |  |  |  |  |  |  |
|  |  |  |  |  |  |  |
|  |  |  | GSD | TSS | URSS | M/LEW |
| Daily Living Skills | F(4, 207.3) = 9.51, p < .001, est. 𝑤^2^ = .073. | SA | t(168) = 5.48, p < .001, *d* = .82 | t(188) = 1.59, p = .506, *d* = .23 | t(190) = 2.30, p = .148, *d* = .33 | t(143) = 4.27, p < .001, *d* = .66 |
|  |  | GSD | - | t(156) = -3.94, p = .001, *d* = .62 | t(170) = -2.72, p < .001, *d* = .41 | t(137)= -.67, p = .963, *d* = .11 |
|  |  | TSS | - | - | t(178) = .87, p = .907, *d* = .13 | t(137) = 2.88, p = .037, *d* = .47 |
|  |  | URSS | - | - | - | t(157) = 1.87, p = .336, *d* = .29 |
|  |  |  |  |  |  |  |
|  |  |  | GSD | TSS | URSS | M/LEW |
| Socialization Skills | F(4, 207.9) = 7.40, p < .001, est. 𝑤^2^ = .056. | SA | t(178) = 5.29, p < .001, *d* = .78 | t(190) = 2.19, p = .186, *d* = .31 | t(201) = 2.75, p = .051, *d* = .39 | t(152) = 3.08, p = .020, *d* = .47 |
|  |  | GSD | - | t(157) = -3.40, p = .007, *d* = .54 | t(170) = -2.48, p = .101, *d* = .38 | t(131) = -1.57, p = .521, *d* = .26 |
|  |  | TSS | - | - | t(180) = .73, p = .950, *d* = .11 | t(131) = 1.29, p = .695, *d* = .21 |
|  |  | URSS | - | - | - | t(146) = .62, p = .972, *d* = .10 |
|  |  |  |  |  |  |  |
|  |  |  |  |  |  |  |
|  |  |  |  |  |  |  |
|  |  |  | GSD | TSS | URSS | M/LEW |
| Movement Skills | F(4, 40.0) = 11.45, p < .001, est. 𝑤^2^ = .260. | SA | t(30.4) = 3.03, p = .037, *d* = .97 | t(58.9) = 1.93, p = .312, *d* = .48 | t(55.9) = 1.63, p = .484, *d* = .43 | t(27.1) = 6.50, p < .001, *d* = 2.11 |
|  |  | GSD | - | t(29.3) = -1.28, p = .702, *d* = .37 | t(35.0) = 10.44, p = .734, *d* = .37 | T(19.0) = 3.24, p = .031, *d* = 1.40 |
|  |  | TSS | - | - | t(60.9) = -.09, p = 1.000, *d* = .02 | t(26.2) = 4.85, p < .001, *d* = 1.39 |
|  |  | URSS | - | - | - | t(31.8) = 4.36, p = .001, *d* = 1.30 |
|  |  |  |  |  |  |  |
|  |  |  |  |  |  |  |
|  |  |  |  |  |  |  |
|  |  |  |  |  |  |  |
|  |  |  |  |  |  |  |
