# Supplementary material for: Exploring sensory phenotypes in autism spectrum disorder
Source: Mol Autism. 2021 Oct 12;12:67. doi: 10.1186/s13229-021-00471-5 (PMC8507349; doi:10.1186/s13229-021-00471-5)
Supplement: Supplementary file 5 — Additional file 5. Full statistics on the one-way ANOVAs conducted to determine whether the RBS-R Repetitive Beahviours subscales differ across the 5 sensory phenotypes are presented. [file 13229_2021_471_MOESM5_ESM.docx]

Supplemental Table E: One way ANOVAs were conducted on the Repetitive Behavior Scales – Revised (RBS-R) scores to determine whether RBS-R subscales differed across the 5 sensory phenotypes (SA – Sensory Adaptive, GSD – Generalized Sensory Differences, TSS – Taste and Smell Sensitivity, URSS – Underresponsive and Sensory Seeking), and M/LEW – Movement and Low Energy / Weakness).

| RBS-R Subscale | ANOVA | Games-Howell Post-Hoc |  |  |  |  |
| --- | --- | --- | --- | --- | --- | --- |
|  |  | Sensory Phenotype | GSD | TSS | URSS | M/LEW |
| RBS-R Total Score | F(4, 260.0) = 58.96, p < .001, est. 𝑤^2^ = .290. | SA | t(135) = -13.7, p < .001, *d* = 1.94 | t(199) = -9.84, p < .001, *d* = 1.25 | t(248) = -5.29, p < .001, *d* = .65 | t(171) = -7.09, p < .001, *d* = .96 |
|  |  | GSD | - | t(186) = 4.27, p < .001, *d* = .60 | t(156) = 9.26, p < .001, *d* = 1.32 | t(168) = 6.84, p < .001, *d* = 1.02 |
|  |  | TSS | - | - | t(222) = 5.08, p < .001, *d* = .65 | t(210) = 2.68, p = .060, *d* = .37 |
|  |  | URSS | - | - | - | t(194) = -2.27, p = .158, *d* = .31 |
|  |  |  |  |  |  |  |
|  |  |  | GSD | TSS | URSS | M/LEW |
| Self-Injury | F(4, 250.8) = 17.62, p < .001, est. 𝑤^2^ = .105. | SA | t(110) = -7.15, p < .001, *d* = 1.04 | t(190) = -5.02, p < .001, *d* = .64 | t(201) = -3.78,  p = .002, *d* = .47 | t(137) = -3.45, p = .007, *d* = .48 |
|  |  | GSD | - | t(152) = 3.33, p = .009, *d* = .48 | t(153) = 4.11, p < .001, *d* = .59 | t(164) = 3.76, p = .002, *d* = .56 |
|  |  | TSS | - | - | t(241) = 1.02, p = .846, *d* = .19 | t(194) = .784, p = .935, *d* = .11 |
|  |  | URSS | - | - | - | t(196) = -.141, p = 1.000, *d* = .02 |
|  |  |  |  |  |  |  |
|  |  |  | GSD | TSS | URSS | M/LEW |
| Stereotypy | F(4, 258.5) = 43.76, p < .001, est. 𝑤^2^ = .232. | SA | t(136) = -10.9, p < .001, *d* = 1.55 | t(195) = -9.51, p < .001, *d* = 1.21 | t(224) = -6.91,  p = .002, *d* = .86 | t(156) = -4.86, p = .007, *d* = .67 |
|  |  | GSD | - | t(190) = 1.92, p = .310, *d* = .27 | t(178) = 4.65, p < .001, *d* = .65 | t(176) = 5.44, p < .001, *d* = .81 |
|  |  | TSS | - | - | t(235) = 2.82, p = .042, *d* = .36 | t(207) = 3.76, p = .002, *d* = .52 |
|  |  | URSS | - | - | - | t(200) = 1.20, p = .749, *d* = .16 |
|  |  |  |  |  |  |  |
|  |  |  | GSD | TSS | URSS | M/LEW |
| Compulsions | F(4, 252.3) = 25.79, p < .001, est. 𝑤^2^ = .149. | SA | t(113) = -8.22, p < .001, *d* = 1.19 | t(182) = -6.89, p = .078, *d* = .88 | t(234) = -4.23, p < .001, *d* = .52 | t(141) = -4.52, p < .001, *d* = .63 |
|  |  | GSD | - | t(166) = 2.61, p = .074, *d* = .37 | t(134) = 5.37, p < .001, *d* = .78 | t(165) = 3.96, p = .001, *d* = .59 |
|  |  | TSS | - | - | T(217) = 3.22, p = .013, *d* = .42 | t(204) = 1.69, p = .440, *d* = .23 |
|  |  | URSS | - | - | - | t(173) = -1.19, p = .758, *d* = .16 |
|  |  |  |  |  |  |  |
|  |  |  | GSD | TSS | URSS | M/LEW |
| Ritualistic / Sameness | F(4, 264.4) = 48.96, p < .001, est. 𝑤^2^ = .253. | SA | t(158) = -12.5, p < .001, *d* = 1.73 | t(224) = -8.74, p < .001, *d* = 1.10 | t(259) = -2.90, p = .033, *d* = .36 | t(195) = -6.44, p < .001, *d* = .86 |
|  |  | GSD | - | t(189) = 4.02, p < .001, *d* = .57 | t(166) = 9.86, p = .074, *d* = 1.40 | t(171) = 6.28, p < .001, *d* = .94 |
|  |  | TSS | - | - | t(228) = 5.99, p < .001, *d* = .77 | t(210) = 2.32, p = .143, *d* = .32 |
|  |  | URSS | - | - | - | t(200) = -3.65, p = .003, *d* = .50 |
|  |  |  |  |  |  |  |
|  |  |  |  |  |  |  |
|  |  |  |  |  |  |  |
|  |  |  |  |  |  |  |
|  |  |  |  |  |  |  |

https://www.socscistatistics.com/effectsize/default3.aspx
